# Recycled, Contaminated, Crumpled Aluminum Foil‐Driven Triboelectric Nanogenerator

**DOI:** 10.1002/advs.202301609

**Published:** 2023-08-06

**Authors:** Jin‐ho Son, Kyunghwan Cha, Seh‐Hoon Chung, Deokjae Heo, Sunghan Kim, Moonhyun Choi, In Soo Park, Jinkee Hong, Sangmin Lee

**Affiliations:** ^1^ School of Mechanical Engineering Chung‐Ang University 84, Heukseok‐ro, Dongjak‐gu Seoul 06974 Republic of Korea; ^2^ Center for Systems Biology Massachusetts General Hospital Boston Massachusetts 02114 USA; ^3^ LS Materials LSMtron Hi‐Tech Center 39, LS‐ro, 116‐gil, Dongan‐gu Anyang‐si Gyeonggi‐do 14118 Republic of Korea; ^4^ Department of Chemical & Biomolecular Engineering College of Engineering Yonsei University 50 Yonsei‐ro, Seodaemun‐gu Seoul 03722 Republic of Korea

**Keywords:** air‐breakdown model, crumpled ball design, recycled foil, self‐powered portable device, triboelectric nanogenerator (TENG)

## Abstract

With rapid urbanization and global population growth, the amount of wasted aluminum foil is significantly increasing. Most deformed and contaminated foil is difficult to recycle; hence, it is landfilled or incinerated, causing environmental pollution. Therefore, using aluminum foil waste for electricity may be conducive to addressing environmental problems. In this regard, various literatures have explored the concept of energy generation using foil, while a crumple ball design for this purpose has not been studied. Thus, a recycled foil‐based crumpled ball triboelectric nanogenerator (RFCB‐TENG) is proposed. The crumpled ball design can minimize the effects of contamination on foil, ensuring efficient power output. Moreover, owing to novel crumpled design, the RFCB‐TENG has some outstanding characteristics to become a sustainable power source, such as ultralight weight, low noise, and high durability. By introducing the air‐breakdown model, the RFCB‐TENG achieved an output peak voltage of 648 V, a current of 8.1 mA cm^3^, and an optimum power of 162.7 mW cm^3^. The structure of the RFCB‐TENG is systemically optimized depending on the design parameters to realize the optimum output performance. Finally, the RFCB‐TENG operated 500 LEDs and 30‐W commercial lamps. This work paves the guideline for effectively fabricating the TENG using waste‐materials while exhibiting outstanding characteristics.

## Graphical abstract

A recycled foil‐based crumpled ball triboelectric nanogenerator (RFBC‐TENG) features ultra‐light weight, low noise, portability, and durability, attributed to its novel crumpled design. By introducing the air‐breakdown model, the device generated a high peak output. The RFBC‐TENG can be a guideline for making high‐power TENGs based on excellent properties as well as solutions for increasing aluminum usage.

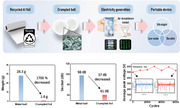

## 1 Introduction

Recently, as modern society has become more industrialized and the global population has exploded, the world faces substantial waste, causing environmental problems, such as soil, air, and water pollution.^[^ ^1^ ^]^ Among the waste materials, aluminum foil (widely used for food, cosmetics, tobacco, and chemical products), owing to its distinctive advantages of cheap, lightweight, good thermal conductivity, impermeability, and broad adaptability, occupies a significant proportion of waste, which is increasing with its usage.^[^ ^2^ ^]^ For example, estimates suggest that, in Europe, almost 860000 tons of aluminum foil are produced annually.^[^ ^3^ ^]^ Furthermore, in China alone, aluminum foil production reached 3.65 Mt in 2017.^[^ ^4^ ^]^ Much aluminum foil is thrown away as waste in a damaged state with contaminants and is challenging to recycle. Accordingly, most foil waste is landfilled or incinerated, resulting in many harmful effects on the natural environment. Converting aluminum foil waste into useful products may provide a new platform to address environmental problems.

In this regard, the triboelectric nanogenerator (TENG), which converts ambient mechanical energy into electricity via contact electrification and electrostatic induction, is a promising option owing to its attractive characteristics, such as simple fabrication,^[^ ^5^ ^]^ broad material availability,^[^ ^6^ ^]^ and versatile operation modes.^[^ ^7^ ^]^ Although some research has been conducted to fabricate the TENG using aluminum foil,^[^ ^8^ ^]^ the conventional structures, especially the plate‐to‐plate type, are disadvantageous for recycled foil because the used aluminum foil is generally torn and wrinkled and commonly thrown away with contaminants. Furthermore, the electrical output performance of the typical plate‐to‐plate type TENGs, which produce electricity through the contact‐separation process of different materials, is significantly affected by the surface state of the materials.^[^ ^9^ ^]^ Thus, deformed, and contaminated foil may negatively affect the output performance. Therefore, a novel structural design strategy that can generate electricity through deformed and contaminated foil is highly desired to employ recycled aluminum foil in the TENG effectively.

In this work, we propose a recycled foil‐based crumpled ball triboelectric nanogenerator (RFCB‐TENG). Aluminum foil, commonly wasted as garbage, is recycled, and crumpled into a ball to generate electricity and power electronics. Owing to the innovative crumpled foil design, the RFCB‐TENG exhibits outstanding characteristics, such as ultralight weight, low noise, and high durability, which are significantly advantageous characteristics for sustainable power sources. The RFCB‐TENG was fabricated using an air‐breakdown model,^[^ ^10^ ^]^ which produces electricity via direct electron transfer in a strong electrical field to generate a high electrical output. Therefore, the RFCB‐TENG achieved a high‐output performance with an output peak voltage of 648 V, a current density of 8.1 mA cm^−3^, and an optimum power density of 162.7 mW cm^−3^. In addition, the structure of the RFCB‐TENG was quantitatively optimized according to various design parameters to maximize the output performance. The optimized RFCB‐TENG demonstrated high performance while operating 500 light‐emitting diodes (LEDs) and 30‐W commercial lamps. This work provides an innovative guideline for fabricating the TENG with outstanding characteristics while not polluting the natural environment.

## 2 Results

### 2.1 Concept and Characteristics of the RFCB‐TENG

**Figure 1:**
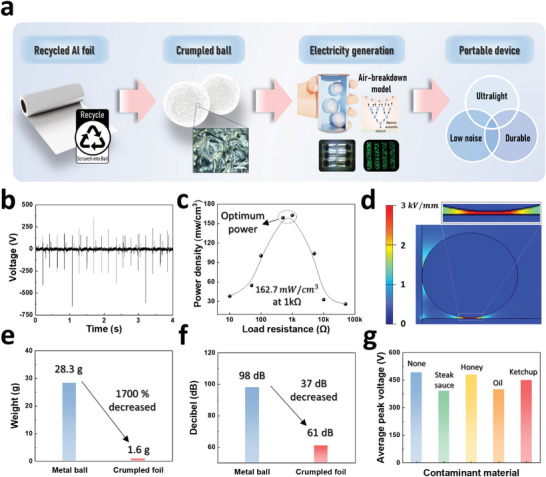
Concept and character of the RFCB‐TENG. a) Schematic process diagram of a portable RFCB‐TENG. b) Voltage output for 4 s. c) Peak power density according to external load resistances. d) Simulation results when the ball touched the PTFE and generated an electrostatic discharge. e) Weight of an equal volume metal ball and crumpled foil. f) Operating decibels for the metal ball and crumpled foil‐based TENGs. g) Average peak voltages of the RFCB‐TENG with kitchen contaminants.

With the global population growth, the volume of wasted aluminum foil (commonly used for foods, cosmetics, and chemical products) is gradually increasing. Most foil waste is eventually landfilled or incinerated, causing many environmental problems. This work proposes a novel strategy for re‐using aluminum foil waste to produce electricity (RFCB‐TENG). **Figure** **1a** presents the broad flow diagram for the concept, fabrication, and application direction of the RFCB‐TENG. The aluminum foil (commonly deformed, contaminated, and thrown away) is recycled by crumpling it into a ball to generate electricity through simple shaking to power commercial electronics. The crumpled ball design and introduction of the air‐breakdown model significantly reduced the effects of the contaminants and deformed structure on the electrical output. The RFCB‐TENG can exhibit a high‐output performance, powering 30‐W commercial lamps and 500 LEDs by simple shaking. Furthermore, the RFCB‐TENG is palm‐sized and highly qualified as a portable power source due to its distinctive characteristics of being ultralight and durable with low noise.

The RFCB‐TENG primarily comprises an acrylic substrate, a charge‐inducing polytetrafluoroethylene (PTFE) layer, aluminum top and bottom electrodes, and crumpled aluminum foil. Figure S1 (Supporting Information) depicts a photograph of the RFCB‐TENG with the dimensions of 50 mm (top and bottom electrode diameter) x 100 mm (device height). Figure 1b and Figure S2 (Supporting Information) display the output voltage and current density of the RFCB‐TENG, respectively. As presented in the graph, the RFCB‐TENG generates an output peak voltage of 648 V and a current of 8.1 mA cm^−3^. The detailed structure and the fabrication process of the RFCB‐TENG are verified in the “Method” section.

In comparison to existing literature on TENG with optimal load resistances within the range of a few megaohms to hundreds of megaohms, our research successfully achieved a significant reduction in the load resistance of the circuit by adopting Air‐breakdown model. The output voltage and current density of the RFCB‐TENG, depending on the external load resistance, were measured to understand the electrical output characteristics, as depicted in Figure S3 (Supporting Information). As the external load resistance increases from 10 to 50 kΩ, the voltage increases from 15.1 to 133.9 V, the current density decreases from 2.5 to 0.2 mA cm^−3^, and the optimum output power of 162.7 mW cm^−3^ is achieved at a load resistance of 1 kΩ (Figure 1c).

**Figure 2:**
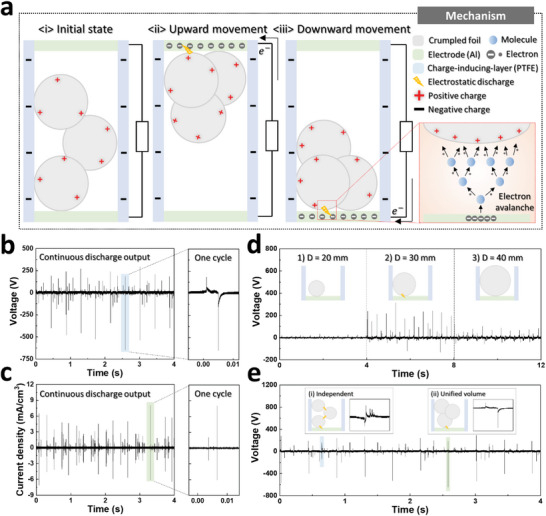
Working mechanism of the RFCB‐TENG. a) Electrical mechanism of the RFCB‐TENG based on the electrostatic discharge. b) Voltage output for 4 s and the magnified graph for one cycle. c) Current density output for 4 s and the magnified graph for one cycle. d) Voltage output depends on the diameter of a single aluminum crumpled ball. e) Voltage outputs with three balls and discharge output when the balls move independently as a unified volume.

Moreover, in this work, the air‐breakdown model, which generates electricity through direct electron transfer in a strong electrical field, was introduced to boost the output performance. According to Paschen's law, the critical breakdown electric field of air is almost 3 kV mm^−1^;^[^ ^11^ ^]^ hence, the simulation result obtained using COMSOL Multiphysics demonstrated that the RFCB‐TENG can successfully induce breakdown and produce a discharge output (Figure 1d). The combination of triboelectrification and electrostatic breakdown in air‐breakdown model based TENG exhibits considerable promise for efficient mechanical energy harvesting. While traditional TENGs of various modes are restricted by the air breakdown effect, which imposes an upper limit on charge density, the air‐breakdown TENG surpasses this limitation. Moreover, it leverages the breakdown effect to its advantage, resulting in a record‐breaking power density among all existing TENGs. It is important to emphasize that the air‐breakdown model‐based TENG demonstrates superior electrical output in comparison to conventional TENG designs.^[^ ^12^ ^]^ The detailed breakdown and discharge output generation mechanisms are presented in **Figure** **2**.

Considering the breakdown principle, a conventional metal ball can also be used to generate a discharge output; however, crumpled foil has several outstanding advantages as a sustainable power source compared with a metal ball. First, the weight of crumpled foil with a diameter of 20 mm is 1.6 g, 17 times less than that of a metal ball (Figure 1e). Next, as shown in Figure 1f, the RFCB‐TENG produced a noise level of 61 dB, signifying a significant reduction compared to the 97 dB emitted when employing a metal ball. This notable decrease in noise can be attributed to the incorporation of an ultralight crumpled foil ball. A noise level of 97 dB is akin to the auditory experience of rock music or a chainsaw, both of which can have detrimental effects on hearing when exposed to continuously. Conversely, a noise level of 61 dB aligns with the typical noise encountered during ordinary conversation. In the context of promoting sustainable device operation, the attainment of this reduced noise level confers substantial advantages. Furthermore, based on its ultralight characteristic, crumpled foil can generate a higher output pulse frequency than a metal ball under the same input. Last, the RFCB‐TENG exhibits remarkable mechanical durability and electrical output reliability. Leveraging these distinctive characteristics, the RFCB‐TENG can sustainably generate electricity and power electronics in portable applications. The photograph and optical microscopic images of the crumpled foil and metal ball are illustrated in Figure S4 (Supporting Information) and describe the surface characteristics of each material. Moreover, the RFCB‐TENG still presents a high‐output performance using crumpled aluminum foil contaminated with various substances (e.g., sauces; Figure 1g).

### 2.2 Working Mechanism of the RFCB‐TENG

**Figure** **2**a presents the schematic diagram for the working mechanism of the RFCB‐TENG during one cycle. The electrical output generation process of the RFCB‐TENG is primarily classified into three parts: the initial state, upward movement, and downward movement. First, at the initial state, the crumpled foil and charge‐inducing layer were charged positively and negatively, respectively, through continuous operation, which is ascribed to the difference in the electron affinity between the two materials. Next, when a driving force is applied, and the positively charged crumpled foil approaches the top electrode, the electrons of the bottom electrode move to the top electrode via electrostatic induction. As the interstitial gap between the crumpled foil and the top electrode decreases, more electrons move to the top electrode and accumulate. Therefore, owing to the oppositely charged crumpled foil and top electrode, a strong electrical field is generated at a very close distance between the two materials.

When the applied electrical field is strong enough to break down the air gap, air breakdown occurs, resulting in the discharge output. The inset schematic diagram in Figure 2a presents the detailed process of the discharge output generation. With a higher electrical field than the critical value of air breakdown, the electrode electrons are directly emitted into the air and collide with air molecules, producing more electrons and positive ions, eventually inducing an electron avalanche phenomenon. A conductive channel is formed through the ionization process, and more direct electron movement achieves a high discharge output. According to Paschen's law, the critical breakdown voltage of air is calculated as follows:^[^ ^13^ ^]^

(1) $$V_{b}=A\frac{\mathrm{Pd}}{\left(\ln\left(\mathrm{Pd}\right)+B\right)}$$

where *A* and *B* are constants related to gas composition and pressure, which are 4.36 × 10^7^ V atm^−1^ m^−1^ and 12.8 in air conditions, respectively. In addition, *d* denotes the gap distance between two materials, and *p* represents the gas pressure, which is 1 × 10^5^ Pa at room temperature.

Last, as the crumpled foil moves to the bottom electrode, it charges positively again through continuous contact with the charge‐inducing layer. Afterward, the same process as in the upward movement is repeated, and a high discharge output is obtained through the air‐breakdown phenomenon. Figure 2b,c displays the continuous discharge output generation and magnified output waveform of the RFCB‐TENG depending on the above mechanism, in which the output peak voltage of 648 V and a current of 8.1 mA cm^–3^ are obtained.

As the electrical output of the RFCB‐TENG can vary with the size of the crumpled foil, the output voltage was measured for several diameters of crumpled foil. As depicted in Figure 2d, it is evident that a diameter of 30 mm generated the highest output voltage because crumpled foil with a diameter of 20 mm cannot fully contact the charge‐inducing layer due to its small size. Accordingly, the device is charged less than it is with a diameter of 30 mm, making it challenging to generate a discharge output, which can also be verified in the simulation results presented in Figure S5 (Supporting Information). In contrast, although the crumpled foil with a diameter of 40 mm can completely contact the charge‐inducing layer, it is too large to move actively between the top and bottom electrodes, causing lower electrical output. Figure 2e illustrates the two different magnified output waveforms of the RFCB‐TENG. As the crumpled foil actively and freely moves upward and downward between the top and bottom electrodes, it can move independently or as a unified volume, resulting in different output waveforms for each case.

### 2.3 Crumpled Ball Optimization

**Figure 3:**
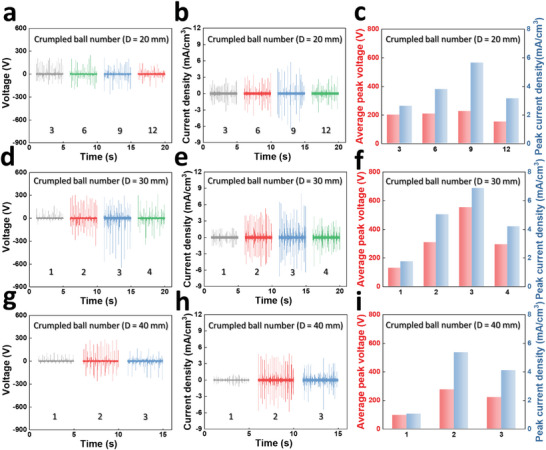
Optimization of the number of balls and their sizes. a) Voltage, b) current density, and c) average peak outputs depending on the number of 20‐mm‐diameter balls. d) Voltage, e) current density, and f) average peak outputs depending on the number of 30‐mm‐diameter balls. g) Voltage, h) current density, and i) average peak outputs depending on the number of 40‐mm‐diameter balls.

Based on the suggested mechanism, **Figure** **3** presents a correlation between the number of crumpled balls and their sizes. A polymethyl methacrylate (PMMA) cylinder with an inner diameter of 46 mm, a thickness of 2 mm, and a height of 100 mm was used as a substrate, and aluminum‐covered PMMA plates sealed the top and bottom of the cylinder used as an electrode. As a charge‐inducing layer, PTFE with a thickness of 0.13 mm was attached to the inner part of the cylinder. For quantitative parameter comparative analysis, we designed a shaker that moves with an amplitude of 25 cm., and its dimensions are presented in Figure S6 (Supporting Information) and device structure illustration is provided in Figure S7 (Supporting Information). The cylinder was operated through a rotational motor‐based shaker, and the rotational speed was controlled by the electrical motor driver and fixed at 140 rpm. Diameters of 20, 30, and 40 mm were used for the crumpled balls, and balls of each size were placed into a cylinder until the height of balls reached 80% of the cylinder.

Figure 3a–c present the electrical output performance of RFCB‐TENG depending on the number of 20‐mm crumpled balls. The average peak voltage of 201.6, 208, 225.6, 151.2 V and the current density of 2.63, 3.78, 5.65, 3.14 mA cm^−3^ were generated when the number of balls was 3, 6, 9, and 12, each. As the number of balls increases from 3 to 9, the contact area between charge inducing layer and the crumpled balls also increases, resulting in enhanced output performances. However, when the number of balls further increases, the excessive occupation of the internal cylinder space causes a lack of movement space, lower contact‐separation process, and eventually reduction of output performances, rather. The corresponding amplified raw output voltage and current graphs according to the number of 20‐mm crumpled balls are provided in Figure S8 (Supporting Information). Next, Figure 3d–f display the electrical output performances of RFCB‐TENG about the number of 30‐mm crumpled balls. For the numbers of 1, 2, 3, and 4, the average peak voltage of 129.6, 308, 552, 293.6 V and current density of 1.75, 5.04, 6.87, 4.20 mA cm^−3^ were yielded, respectively. The optimum output was achieved at the number of 3, which is the result of trade‐off between the increased contact area and reduction of active volume as similar as the case of 20‐mm crumpled balls. Meanwhile, the overall electrical output performances was enhanced when compared with the case of 20‐mm crumpled balls. The magnified raw output voltage and current graphs for the number of 30‐mm crumpled balls are demonstrated in Figure S9 (Supporting Information). Last, Figure 3g–i show the electrical performance depending on the number of 40‐mm balls. RFCB‐TENG with 1, 2, and 3 crumpled balls generated an average peak voltage of 96.8, 274.4, 220 V and current density of 1.04, 5.37, 4.10 mA cm^−3^, respectively. The overall output performances was reduced when compared with the case of 30‐mm. This is because that the size increment results in a decreased horizontal movement space. Particularly, a 40‐mm foil ball in a cylinder with an inside diameter of 42 mm significantly restricts horizontal movement, and it is difficult to achieve sufficient speed to increase the contact force with the charge‐inducing layer, leading to a lower output than the case of the number of 30‐mm balls. The amplified raw output voltage and current graph of the RFCB‐TENG for the number of 40‐mm balls are presented in Figure S10 (Supporting Information). Eventually, optimal value was achieved when the piled‐up crumpled balls reached 60% of the height standard of the cylinder, with 9 balls for a 20 mm diameter, 3 balls for a 30 mm diameter, and 2 balls for a 40 mm diameter, and RFCB‐TENG with three 30‐mm balls exhibited the optimum electrical output performances and utilized in the following experiments.

### 2.4 Output Performance Measurements of the RFCB‐TENG

**Figure 4:**
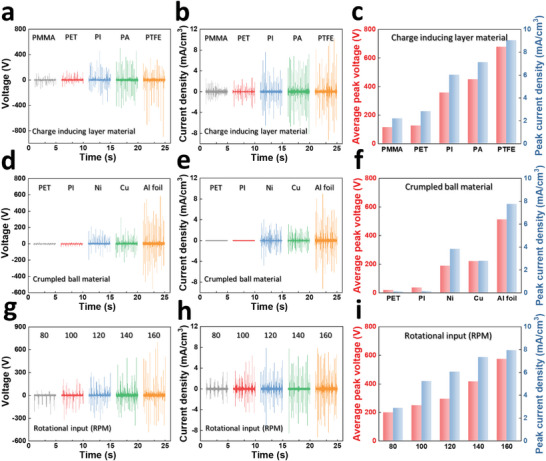
Parametric analysis of the designed variables of the RFCB‐TENG. a) Voltage, b) current density, and c) average peak outputs depending on various charge‐inducing layer materials. d) Voltage, e) current density, and f) average peak outputs depending on various crumpled ball materials. g) Voltage, h) current density, and i) average peak outputs depending on various rotational inputs.

To verify the output performance of the RFCB‐TENG, we conducted the electrical output measurement for various parameters including charge inducing layer material, crumpled ball material, and rotational input speed. The structure and design variables of RFCB‐TENG are illustrated in Figure S7 (Supporting Information) and the detailed fabrication process is presented in “Method” section. First, **Figure** **4a** **–** **c** present the voltage, current density, and average peak voltage/current density of RFCB‐TENG about various charge inducing layer materials at a 140 RPM. The average peak voltage of 113.6, 125.6, 256.8, 448.8, 678.4 V and current density of 2.18, 2.83, 6.00, 7.12, 9.04 mA cm^−3^ were obtained for PMMA, polyethylene terephthalate (PET), polyimide (PI), polyamide (PA), and PTFE, respectively. Based on Paschen's law, the electrostatic discharge output is relevant to the intensity of electrical field between the electrode and crumpled ball. As the ball material is fixed as aluminum foil, the charge inducing layer material is the dominant factor to determine the output performance. As shown in the graph, the optimum output was obtained when the charge inducing layer was PTFE, and followed by PA, PI, PET, and PMMA. This tendency is consistent with the 2 between each material and aluminum, which can also be verified in well‐known triboelectric series.^[^ ^14^ ^]^ Accordingly, PTFE was selected as a charge inducing layer material in the following experiments. The corresponding magnified raw voltage and current plot for charge‐inducing‐layer are demonstrated in Figure S11 (Supporting Information).

Next, considering our suggested discharge output generation mechanism, the crumpled ball material is significantly important parameter to optimize the output performance. Figure 4d–f depicts the electrical output performance of RFCB‐TENG for several non‐conductive materials (PTFE, PI) and conductive materials (nickel [Ni], copper [Cu], Al foil) at a 140 RPM. For PET, PI, Ni, Cu, and Al foil, the peak voltage of 16.72, 33.84, 186, 220.4, 551.2 V and current density of 0.08, 0.11, 3.82, 2.76, 7.75 mA cm^−3^ were generated, respectively. Considering above suggested discharge generation mechanism, the non‐conductive materials (PET, PI) are hard to generate electrostatic discharge. Accordingly, the non‐conductive materials generated low electrical output performance based on the conventional electrostatic induction. In contrast, the conductive materials (Ni, Cu, Al foil) can generate the electrostatic discharge output, which causes the higher output performances than non‐conductive materials. Meanwhile, among them, aluminum foil‐based crumped balls yielded the highest electrical output owing to the largest electron affinity difference with PTFE layer.^[^ ^13^, ^14^ ^]^ The magnified plots of voltage and current output depending on the different crumpled ball materials are presented in Figure S12 (Supporting Information).

Furthermore, the effect of the external force on the electrical output was also verified according to the input RPM. Figure 4g–i shows the electrical performance of the RFCB‐TENG for different revolutions per minute (RPM). For 80, 100, 120, 140, and 160 RPM, RFCB‐TENG produced the average peak voltages of 197.2, 248.4, 294, 416.8, 573.6 V and current density of 2.86, 5.22, 6.06, 7.31, 7.94 mA cm^−3^, respectively. It is obvious that as the input RPM increases, the output performance of RFCB‐TENG also increases. This is attributed to the increased contact force between the charge‐inducing layer and crumpled balls, resulting in an increase in the electrical output peak values as well as output frequency. The corresponding magnified raw output voltage and current graphs under different RPM are provided in Figure S13 (Supporting Information).

### 2.5 Durability and Applications of the RFCB‐TENG

**Figure 5:**
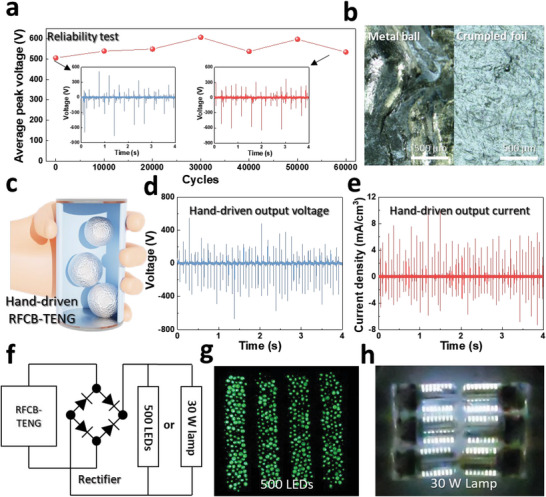
Durability test and application with a hand‐driven RFCB‐TENG. a) Average peak voltage for long working cycles in a magnified graph at the beginning and end. b) 250X magnified photograph of electrodes that operated 60000 cycles with metal and crumpled balls. c) Illustration of a hand‐driven RFCB‐TENG. d) Voltage and e) current density output of a hand‐driven RFCB‐TENG. f) Electrical circuit to power 500 LEDs or a 30‐W lamp. Photograph of g) 500 LEDs and h) a commercial 30‐W lamp powered by a hand‐driven RFCB‐TENG.

In addition, we conducted studies on the practical use of an optimally designed device. The mechanical durability and electrical output stability of an energy harvester is essential for real‐world applications. In this respect, the low‐weight properties of a crumpled aluminum ball could increase the mechanical and electrical continuance of the device. As indicated in **Figure** **5a** and Figure S14 (Supporting Information), a long‐term electrical sustainability test for the RFCB‐TENG was conducted, revealing that a high electrical output was constant during the device operation for 60000 cycles under 140 rpm of external load. Furthermore, the result of the mechanical durability test is provided in Figure 5b and Figure S15 (Supporting Information). Figure 5b captures the magnified device electrode surfaces operated with the metal balls and crumpled foil for 60000 cycles, respectively. Deep cracks and tearing were observed on the electrode surface colliding with the steel ball, due to the greater impact from the heavy weight. On the other hands, the crumpled foil has only caused a few scratches and dimples compared to the initial state presented in Figure S16 (Supporting Information). Moreover, this difference in damage is illustrated in the overall photograph of the electrodes (Figure S15, Supporting Information). In addition to these strengths of durability, the advantages of low noise and an ultralight weight (Figure 1e,f) facilitate the use of the RFCB‐TENG as a hand‐operated device. Figure 5c illustrates a hand‐driven RFCB‐TENG, and a photograph is provided in Figure S17 (Supporting Information). Figure 5d,e reveals the electrical hand‐driven output of the RFCB‐TENG and its capability to generate a high frequency and output through human force. Figure 5f depicts a single full‐bridge rectifying electrical circuit to turn on the LEDs or commercial lamps. Figure 5g and Video S1 (Supporting Information) demonstrate that the optimized RFCB‐TENG can power 500 LEDs with high frequency through hand operation. Furthermore, six serially connected G9 lamps (one lamp has a rated power of 5 W) totaling 30 W can also be ed powered by a hand‐operated RFCB‐TENG (Figure 5e; Video S2, Supporting Information). Additionally, commercial 10, 22, 47, and 100 µF capacitors were charged to 1.6, 1.24, 0.84 and 0.56 V, respectively, for 200 s of hand‐operation. (Figure S18, Supporting Information) Thus, we propose a long‐life, high‐output, hand‐operatable, eco‐friendly power source via aluminum foil waste and anticipate that this device will be considered a basic energy‐from‐waste strategies.

## 3 Conclusion

In summary, we developed an RFCB‐TENG, which has distinctive merits of being ultralight, and highly durable with low noise. Here, aluminum foil waste was recycled, crumpled into a ball shape, and used for producing electricity. RFCB‐TENG introduced the air‐breakdown model to achieve high output performance. Owing to the electrostatic discharge‐based mechanism, the RFCB‐TENG realized high electrical output with a peak voltage of 648 V, a current of 8.1 mA cm^−3^, and an optimum power of 162.7 mW cm^−3^. The structure of RFCB‐TENG was quantitatively optimized for several design parameters to obtain the maximum electrical output. Based on the ultralight characteristic, RFCB‐TENG demonstrated its mechanical durability and electrical output stability during 60000 cycles, which facilitated its application as portable power source. Finally, RFCB‐TENG turned on 500 LEDs and 30‐W commercial lamps by simple hand operation. This work suggested a novel strategy for fabricating nanogenerator based on recycled materials while realizing remarkable output performances and good characteristics as portable power source.

## 4 Experimental Section

### Fabrication of the RFCB‐TENG

The RFCB‐TENG comprises a PMMA substrate, charge‐inducing layer, crumpled ball, and electrode. The inner diameter of the cylinder was 46 mm, and the outer diameter was 50 mm with a height of 10 cm. Commercial PTFE tape with a thickness of 0.13 mm (ASF‐110, Chukoh Chemical Industries Co., Japan) was attached to the interior area of the cylinder as a charge‐inducing layer. Aluminum foil with a thickness of 15 µm (Hwami Co, South Korea) was used as a crumpled ball material. To check the effect of kitchen contaminants on electrical output, commercial sauces were evenly spread on the foils, and after few minutes, contaminated foils were crumpled into balls. As an electrode part, aluminum tape with a thickness of 0.05 mm (Duksung Hitech Co., South Korea) was pasted over an area 2 mm thick with a 5‐cm‐diameter round acrylic substrate, and the upper and lower ends of the cylinder were sealed. The 25 cm of vertical movement was applied by a rotational motor‐based shaker (Motor: GBM‐02JSK11, GR Electronics, South Korea; Gear head: S9KC10BH, SPG Co., South Korea), and the speed was controlled by a motor driver (BX‐3000A, GR electronics, South Korea).

### Electrical Measurement and Characterization

With a mixed‐domain oscilloscope (MDO 34, Tektronix Co., United States), the differential probe (THDP0100, internal resistance: 10 MΩ, Tektronix Co., United States) and current probe (TCP0030A, Tektronix Co., United States) were used to measure the output voltage and current.

## Conflict of Interest

The authors declare no conflict of interest.

## Supporting information

Supporting Information

Supplemental Video 1

Supplemental Video 2

## Data Availability

The data that support the findings of this study are available in the supplementary material of this article.
