# Supplementary material for: Recycled, Contaminated, Crumpled Aluminum Foil‐Driven Triboelectric Nanogenerator
Source: Adv Sci (Weinh). 2023 Aug 6;10(28):2301609. doi: 10.1002/advs.202301609 (PMC10558650; doi:10.1002/advs.202301609)
Supplement: Supplementary file 1 — Supporting Information [file ADVS-10-2301609-s002.pdf]

ADVANCED SCIENCE
Open Access

Supporting Information

for *Adv. Sci.*, DOI 10.1002/advs.202301609

Recycled, Contaminated, Crumpled Aluminum Foil-Driven Triboelectric Nanogenerator

Jin-ho Son, Kyunghwan Cha, Seh-Hoon Chung, Deokjae Heo, Sunghan Kim, Moonhyun Choi, In Soo Park, Jinkee Hong and Sangmin Lee**

Recycled, contaminated, crumpled aluminum foil-driven triboelectric nanogenerator

Jin-ho Son[1,†], Kyunghwan Cha[1,†], Seh-Hoon Chung[1], Deokjae Heo[1], Sunghan Kim[1], Moonhyun Choi[2], Park In Soo[3], Jinkee Hong[4,], Sangmin Lee[1,*]*

[1] School of Mechanical Engineering, Chung-Ang University, 84, Heukseok-ro, Dongjak-gu, Seoul, Republic of Korea

[2] Center for Systems Biology, Massachusetts General Hospital, Boston, Massachusetts 02114, United States

[3] LSMtron Hi-tech center, 39, LS-ro, 116-gil, Dongan-gu, Anyang-si, Gyeonggi-do, 14118, Korea

[4] Department of Chemical & Biomolecular Engineering, College of Engineering, Yonsei University, 50 Yonsei-ro, Seodaemun-gu, Seoul 03722, Republic of Korea

[†] These authors contributed equally to this work.

*Corresponding authors

*Email: Sangmin Lee (slee98@cau.ac.kr)

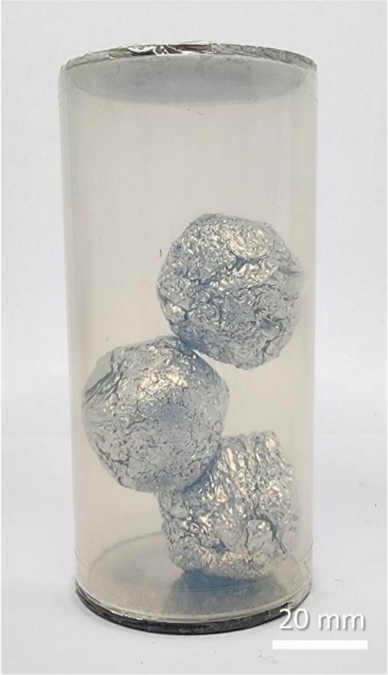

Supporting Information 1| Photograph of RFCB-TENG device.

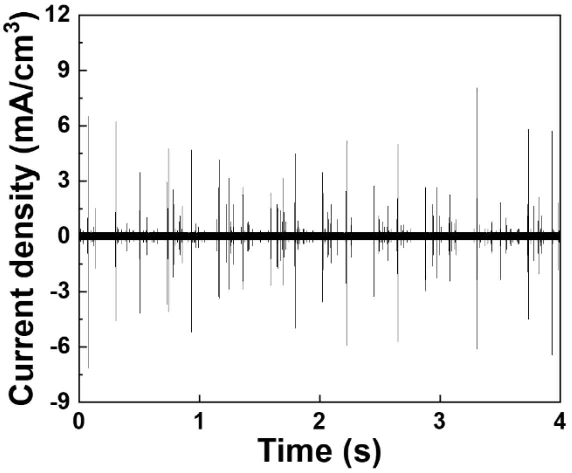

Supporting Information 2| Current density output of RFCB-TENG.

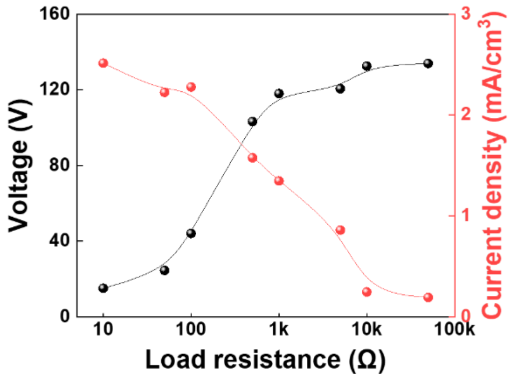

Supporting Information 3| Peak average voltage and peak average current density plot of RFCB-TENG depending on the external load resistance.

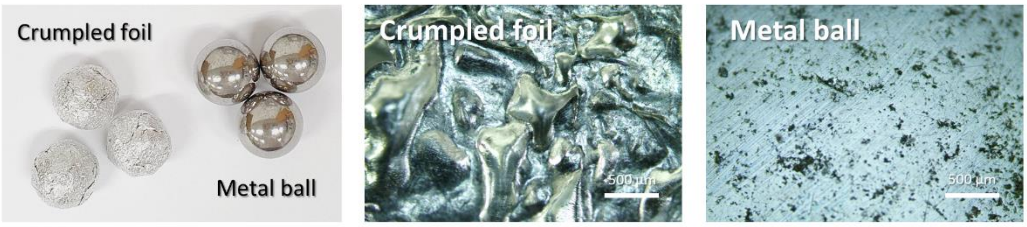

Supporting Information 4| General and 250x magnified pictures of metal balls and crumbled balls.

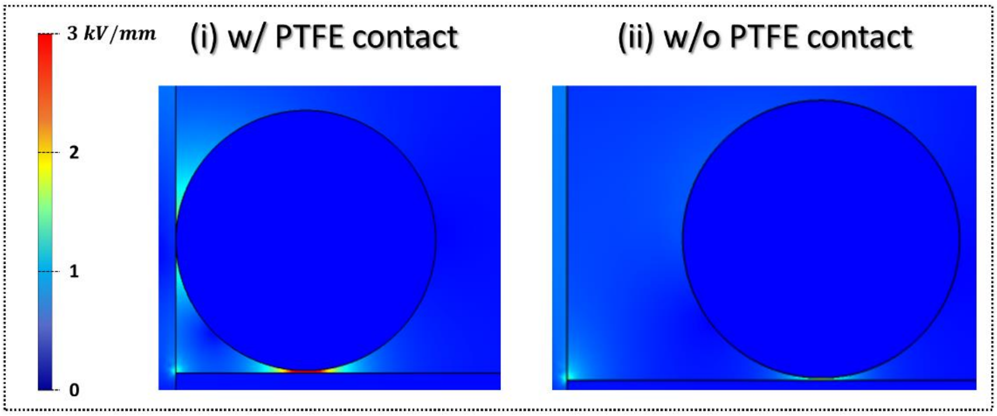

Supporting Information 5| COMSOL multiphysics simulation results with or without contact between PTFE and crumpled balls.

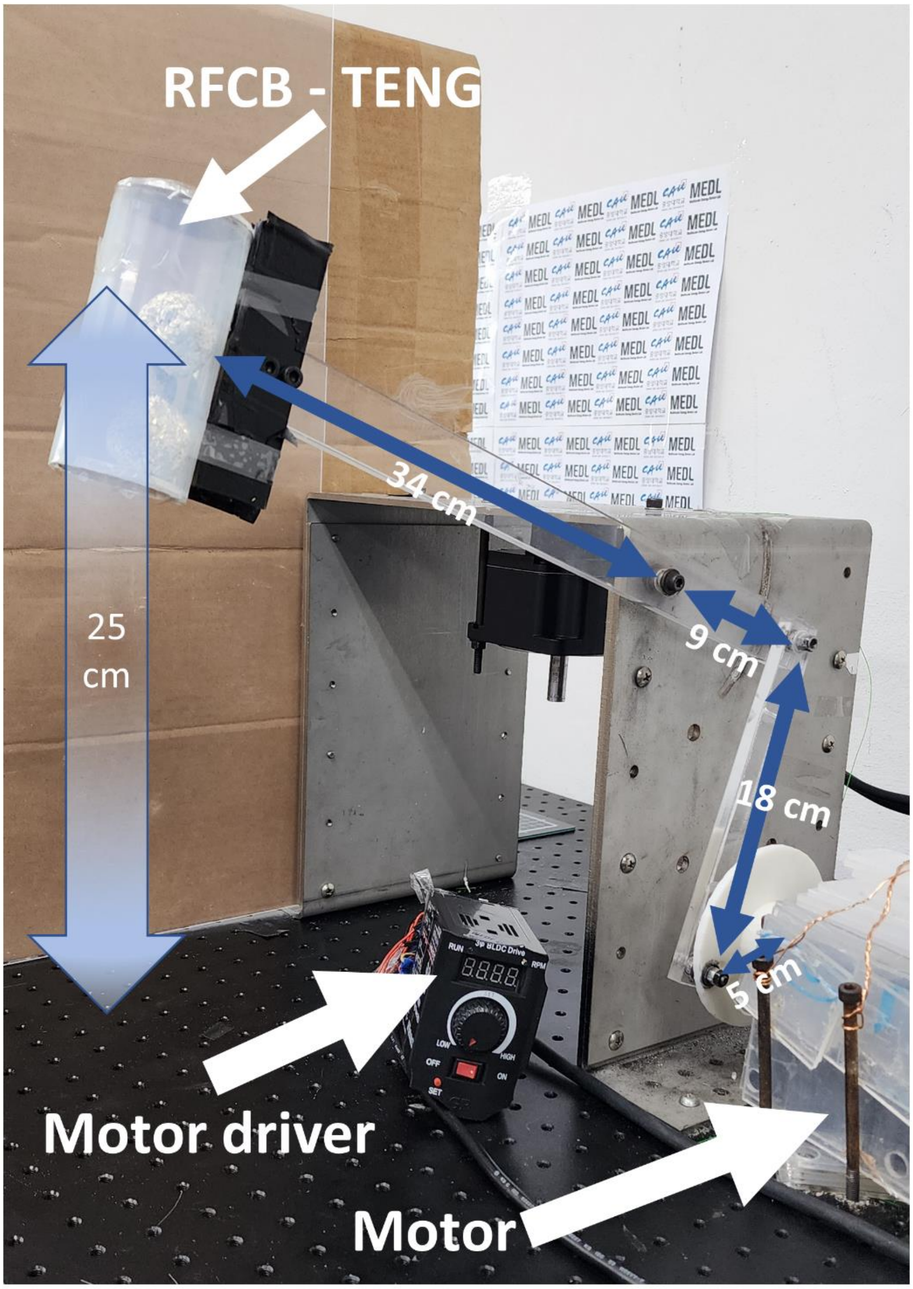

Supporting Information 6| Photograph and demension of shaker used for mechanical input.

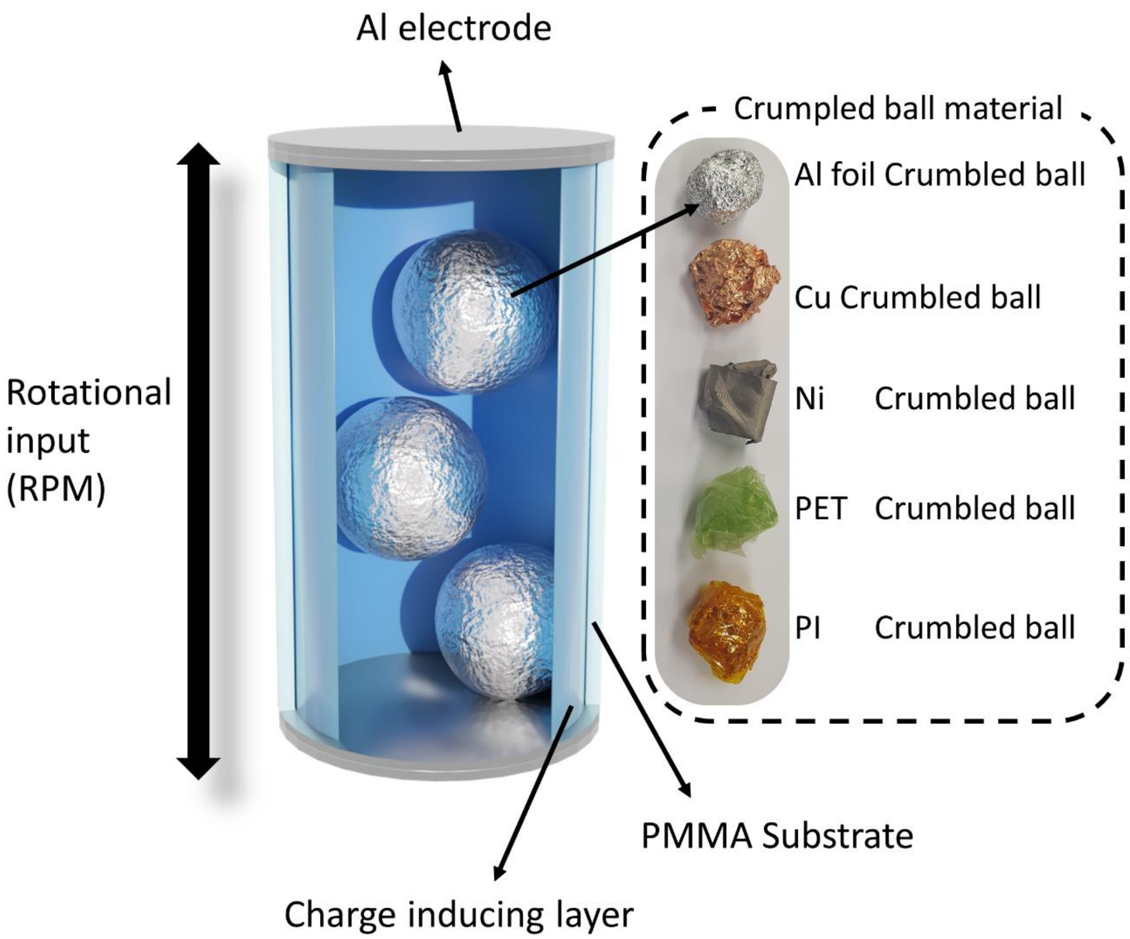

Supporting Information 7| Schematic of RFCB-TENG with designed parameters.

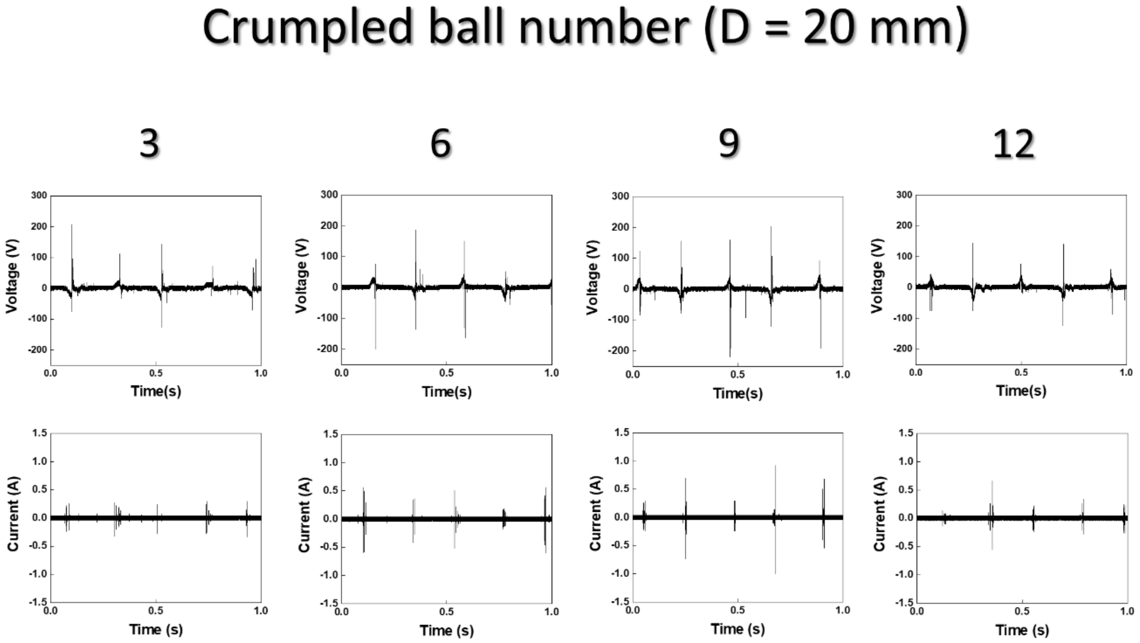

Supporting Information 8| Magnified plots of voltage and current output depending on the number of 20 mm crumpled balls.

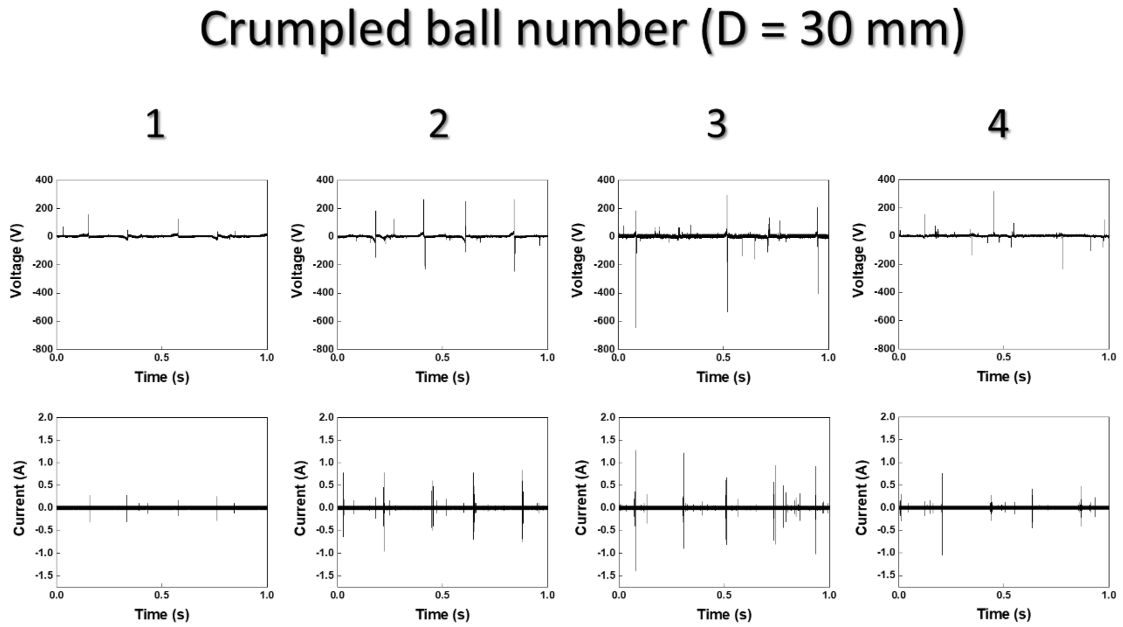

Supporting Information 9| Magnified plots of voltage and current output depending on the number of 30 mm crumpled balls.

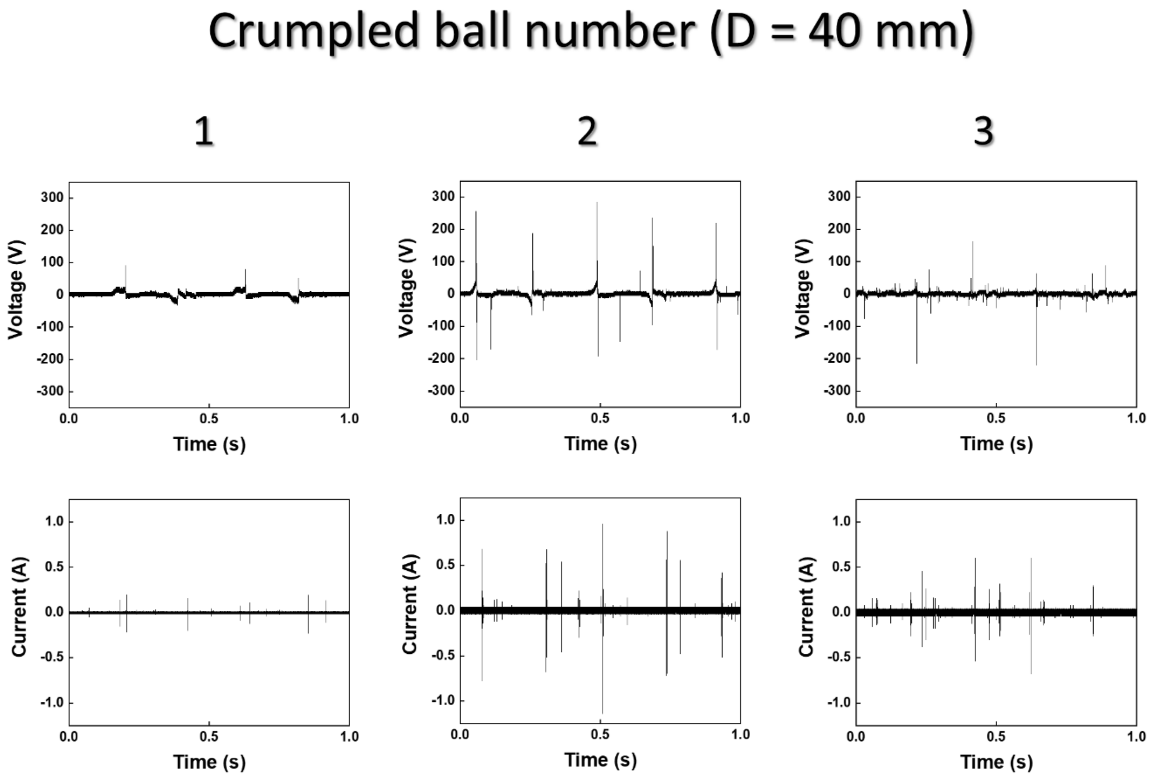

Supporting Information 10| Magnified plots of voltage and current output depending on the number of 40 mm crumpled balls.

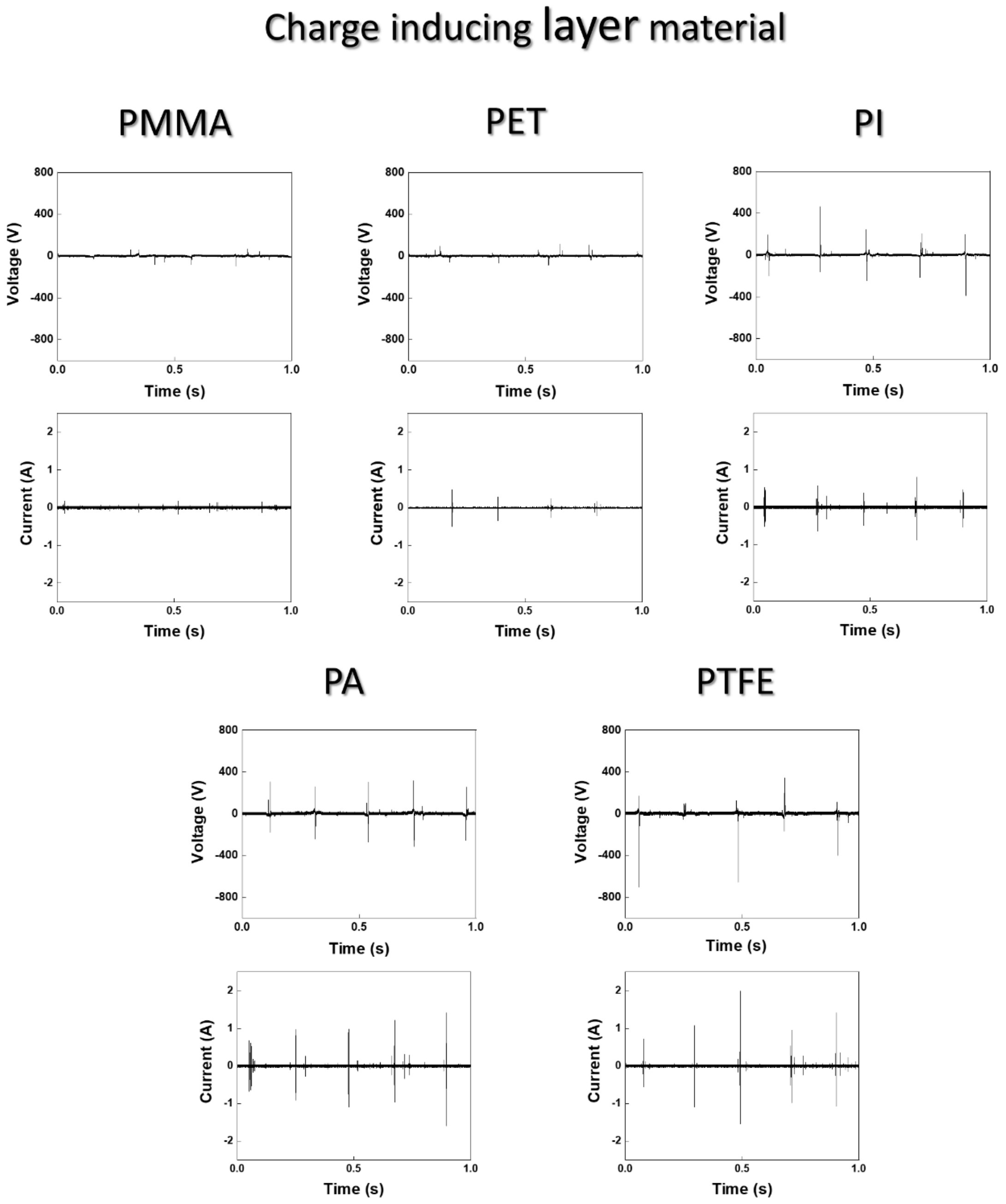

Supporting Information 11| Magnified plots of voltage and current output depending on the different layer materials.

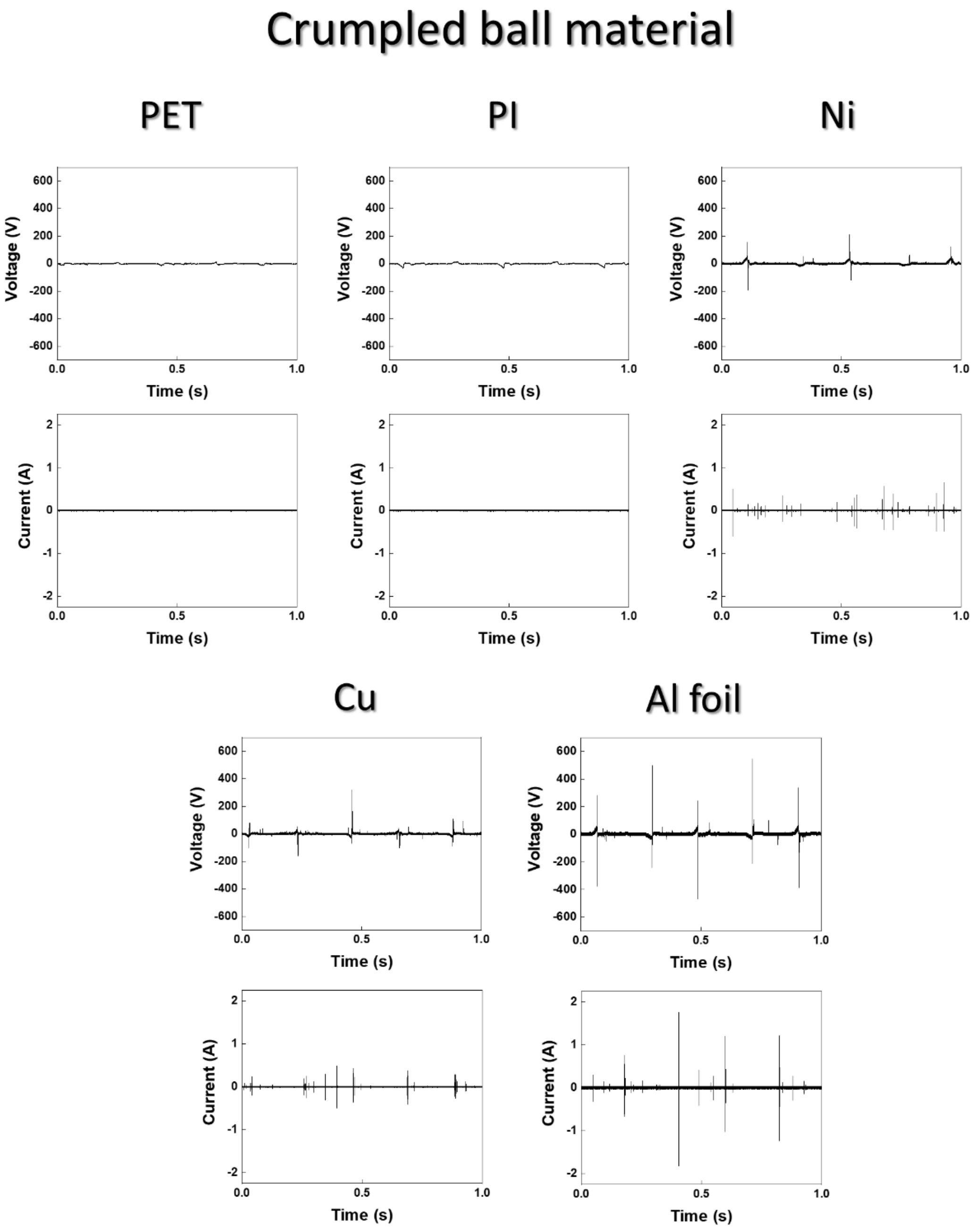

Supporting Information 12| Magnified plots of voltage and current output depending on the different crumpled ball materials.

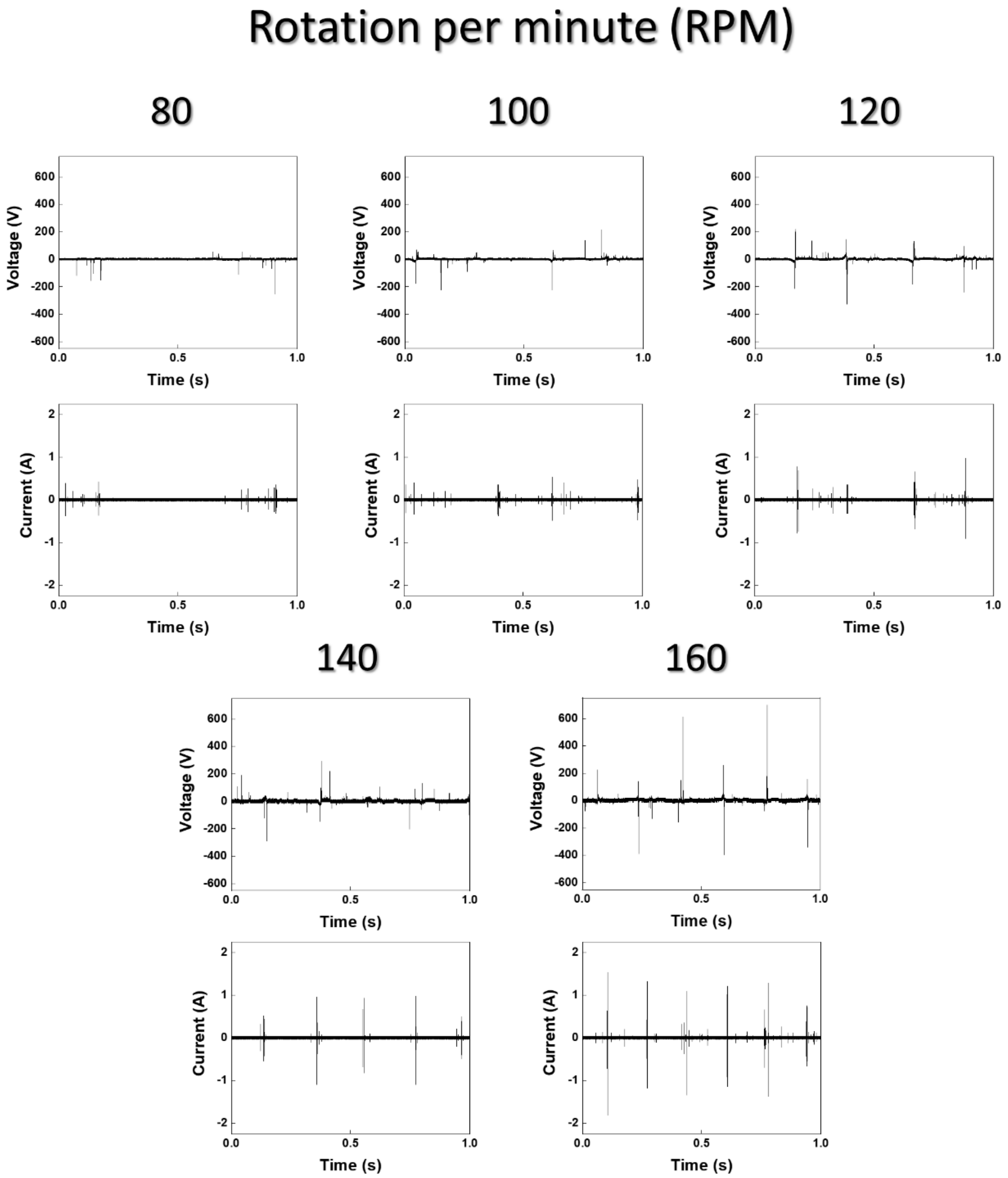

Supporting Information 13| Magnified plots of voltage and current output depending on the different input rotational speed (RPM).

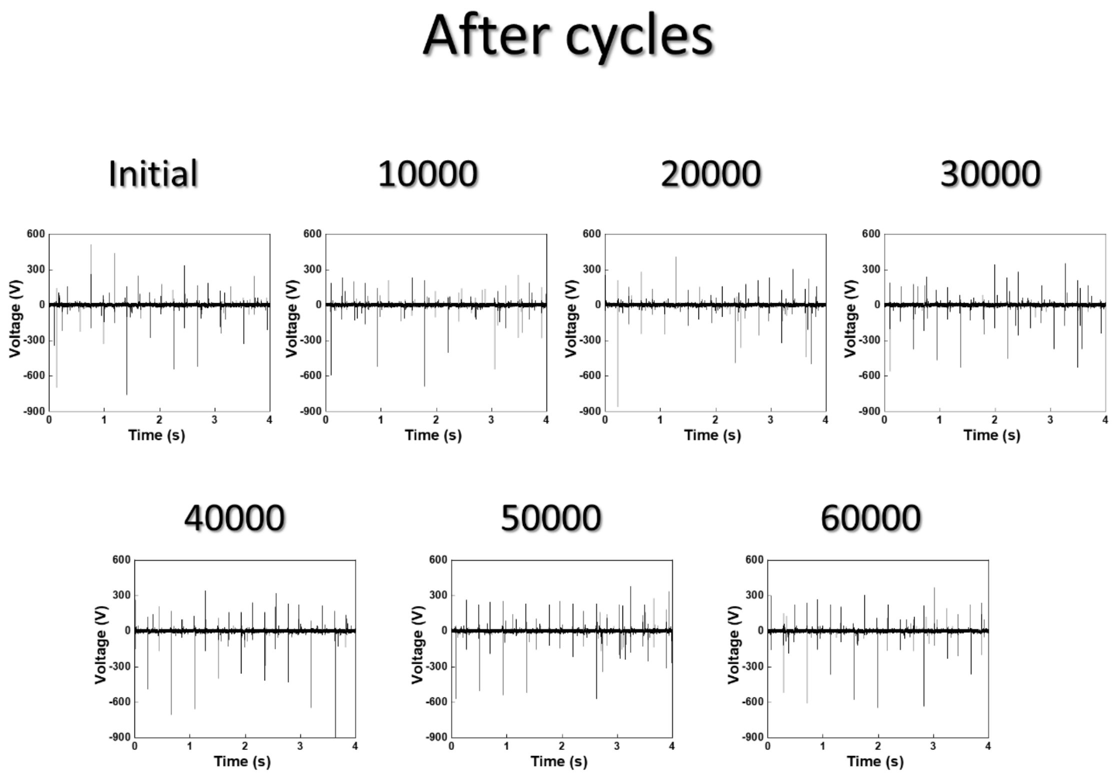

Supporting Information 14| Voltage output of RFCB-TENG during long-operating cycles.

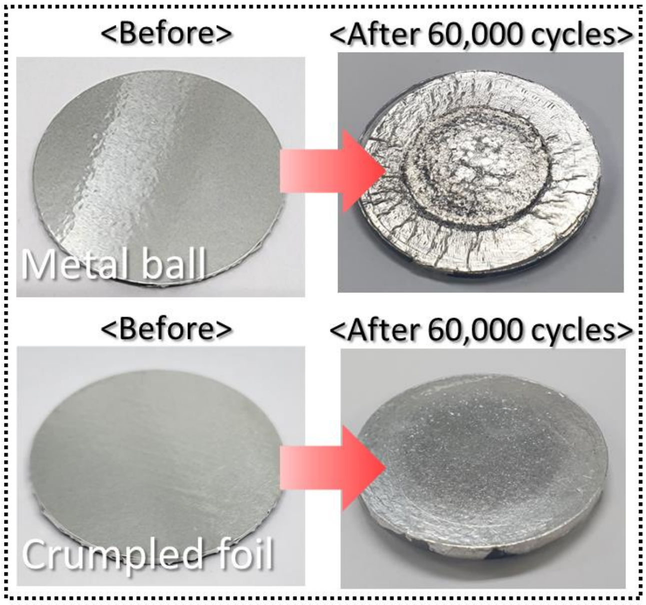

Supporting Information 15| Before and after photographs of the electrodes used in aluminium balls and crumpled foil-based RFCB-TENGs, respectively, during 60000 cycles.

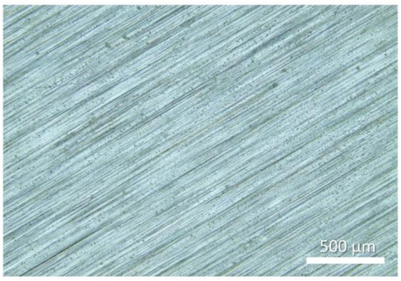

Supporting Information 16| 250X magnified photograph of aluminium electrode surface before device operation.

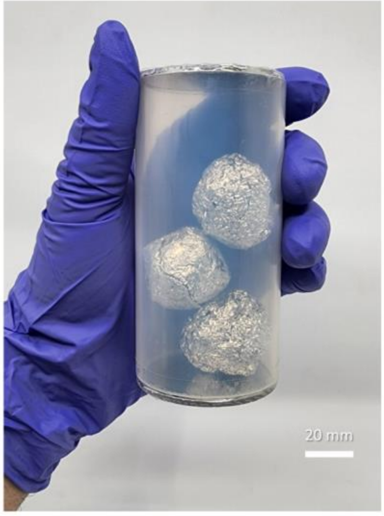

Supporting Information 17| Photograph of hand-driven RFCB-TENG

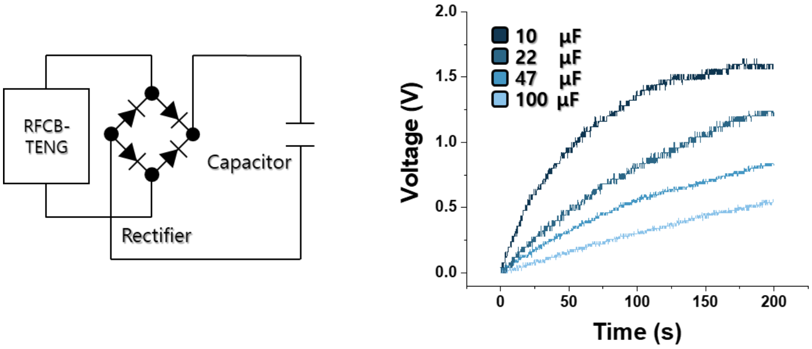

Supporting Information 18| Circuit and charge graph for charging a commercial capacitor

Supporting Video 1| RFCB-TENG lighting 500 LEDs with hand-operation

Supporting Video 2| RFCB-TENG operating 30W G9 lamps with hand-operation
